# A step forward for an attenuated blood-stage malaria vaccine

**DOI:** 10.1186/s12916-018-1197-1

**Published:** 2018-11-09

**Authors:** James M. Burns

**Affiliations:** Department of Microbiology and Immunology, Center for Molecular Parasitology, Drexel University College of Medicine, 2900 Queen Lane, Philadelphia, PA 19129 USA

**Keywords:** Attenuated vaccines, Blood-stage parasites, *Plasmodium falciparum*, Malaria

## Abstract

Efforts to develop an effective malaria vaccine have encountered multiple challenges, and have had limited success to date. As the need remains urgent, novel approaches must be explored. One concept that has gained attention uses whole malaria parasites. Building on preclinical studies in animal models, Stanisic et al. describe the development of a vaccine based on chemically attenuated *Plasmodium falciparum* blood-stage parasites, with an evaluation of safety and immunogenicity in malaria-naïve human subjects. The vaccine was shown to be safe, well tolerated, and capable of priming antigen-specific T cells. This work, and the completion of an initial clinical trial in human subjects, represents a significant advance. While the path forward for this attenuated vaccine remains challenging, these initial findings are encouraging. Importantly, the results provide the foundation and framework for testing modified immunization protocols, and designing subsequent clinical trials to further evaluate safety, test for enhanced immunogenicity, and ultimately measure protective efficacy.

Please see related article: https://bmcmedicine.biomedcentral.com/articles/10.1186/s12916-018-1173-9

## The promise of whole parasite vaccines

Since the early 2000s, concerted control efforts have resulted in a marked reduction in malaria morbidity and mortality [1]. Nevertheless, the global burden of malaria remains unacceptably high. Many believe that integration of an effective malaria vaccine into control programs will be critical for success [2]. The decades-long effort to develop a malaria vaccine has enhanced our understanding of protective immune mechanisms and targets. Although several different subunit malaria vaccines have progressed to clinical testing, the results have generally been disappointing [3–8]. Issues of limited efficacy, short durability of protection, and/or antigen polymorphism, have reduced enthusiasm to advance most of these candidates. These challenges have been particularly problematic for subunit vaccines targeting blood-stage parasites. Given the need to further reduce the burden of malaria, all approaches, traditional and non-traditional, should be considered and evaluated.

The development of whole-parasite vaccines targeting pre-erythrocytic-stage malaria parasites (sporozoites, liver-stage parasite) has gained prominence as an alternate to subunit vaccines [2]. Concepts include radiation-attenuated [9] or genetically attenuated [10] parasites, as well as infection with non-attenuated sporozoites with concurrent antimalarial drug administration [11]. While challenges remain in the development of these vaccines, encouraging initial clinical trial results are driving the effort. Key advantages of an attenuated vaccine approach include limited replication of parasites displaying a large array of antigens, with the potential to elicit protection that is not dependent on a single antigen, and/or a single immune effector mechanism. Michael Good and collaborators have adapted this concept, and evaluated the feasibility of developing a chemically attenuated whole-parasite vaccine targeting blood-stage malaria parasites [12, 13]. The results from these animal model system studies have been very encouraging, with solid and durable protection achieved.

## Addressing the challenges for development

Stanisic et al. [14] present a pilot study evaluating the safety and immunogenicity of a chemically attenuated *Plasmodium falciparum* asexual blood-stage vaccine in malaria-naïve human subjects. This is the first clinical study of its kind. Moving this vaccine concept forward to testing in human subjects represents a significant advance. This initial effort focused on four key questions. First, can this vaccine be manufactured in compliance with regulatory standards? The group built on prior successes in which *P. falciparum*-infected erythrocytes were generated under conditions suitable for approval of trials involving infection of human subjects. Unique to the current study was the need to monitor the residual level of the drug used for attenuation (tafuramycin-A) in the final vaccine formulation, given the drug’s genotoxic potential. Residual drug concentrations well below threshold values of concern were observed. These initial results are very encouraging, but this matter will likely attract additional scrutiny as trials progress.

Second, can *P. falciparum*-parasitized erythrocytes be chemically treated in vitro to ensure no breakthrough infection in vivo, while maintaining the required level of viability? The answer appears to be yes. While breakthrough infections were noted with parasites treated with a low concentration of the drug, a four-fold higher concentration effectively achieved attenuation.

Third, is the vaccine safe? Safety was carefully monitored for a 90-day period. In routine assessments in volunteers who received the fully attenuated vaccine, no issues of concern were observed. The one exception of note was the detection of antibodies to a minor red blood cell (RBC) antigen in one subject. This prompted the team to produce attenuated vaccines for the remaining volunteers using their own RBCs. This is not a viable solution for vaccine manufacture, so this matter will need to be closely monitored moving forward. If the problem persists, further development of this attenuated vaccine will be in jeopardy.

The fourth question is particularly important: is the vaccine immunogenic? Considering the novelty of the vaccine design and potential safety issues, this first trial – by necessity – included a small number of subjects who received only one dose of attenuated *P. falciparum*-parasitized RBCs. As such, it was not unexpected to see heterogeneity in immune responses. The interpretation of immunogenicity data was also limited, to some degree, by the responsiveness of peripheral blood mononuclear cells from these malaria-naïve volunteers to in vitro stimulation with *P. falciparum*-infected RBCs at baseline. Despite these issues, it was notable that immunization with the fully attenuated vaccine primed antigen-specific T cells to proliferate and produce TNFα and IFNγ. These results are encouraging because these two cytokines contribute to protection against blood-stage parasites when their production is appropriately regulated.

Also of interest was the lack of detectable, antigen-specific antibody responses in this cohort. This may be attributed to the administration of a single priming dose. However, data from studies in naïve animals, which go back as far back as 1981 [15], and through the recent work of Good and colleagues with chemically attenuated *P. chabaudi* blood-stage vaccines [12], indicate that CD4+ T cell responses can contribute to protection, independent of antibody. Induction of cell-mediated immune mechanisms in human subjects by administration of an attenuated blood-stage vaccine would add a new dimension to the malaria vaccine effort. Current blood-stage vaccines almost exclusively target the production of parasite-neutralizing antibodies.

## Conclusions

This work is in an early phase. The group passed some significant hurdles in completing this initial testing in human subjects. Similar to whole-parasite pre-erythrocytic-stage vaccines, issues related to the scalability of production, and logistics of storage and distribution will need to be addressed at some point. Other questions require more immediate attention. Is the short period in which attenuated parasites persist in vivo in human subjects adequate to drive robust immune responses? Can immunogenicity be improved with booster immunizations without compromising safety, and without exacerbating the problem of the induction of anti-RBC antibodies? Does the vaccine induce responses to a breadth of blood-stage antigens as hoped, or only to selected, immunodominant and/or polymorphic antigens? Are vaccine-induced responses adequately protective? This last question represents the most significant challenge. In endemic areas, natural immunity to blood-stage malaria parasites does not typically develop after a single *P. falciparum* infection [16]. As such, induction of strain-transcending protection by infection with attenuated *P. falciparum*-parasitized RBCs would be fundamentally different.

While the path forward may be challenging, this trial represents a major step. The results provide the foundation and framework to design subsequent trials with larger cohorts. These key questions can now be systematically addressed. Additional study of this vaccine will almost certainly expand our understanding of protective immunity to blood-stage malaria, and inform the efforts of the wider community working to develop a vaccine for malaria.
